# One Health: People, Animals, and the Environment

**DOI:** 10.3201/eid2204.151887

**Published:** 2016-04

**Authors:** Casey Barton Behravesh

**Affiliations:** Centers for Disease Control and Prevention, Atlanta, Georgia, USA

**Keywords:** One Health, humans, animals, environment, zoonoses

The One Health concept recognizes that the health of humans is connected to the health of animals and the environment. An interdisciplinary One Health approach involving human, animal, and environmental health partners worldwide is critical to address current public health issues, which include emerging infectious and zoonotic diseases. The book One Health: People, Animals, and the Environment reviews core concepts of One Health and highlights key One Health issues of public health importance.

The book comprises 5 sections. The first section covers the need for a One Health approach and reasons such an approach is important. Topics include the human–animal interface, ecologic approaches to studying zoonoses, and the role of wildlife in emerging infectious diseases. The second section covers zoonotic and environmental drivers of emerging infectious diseases. This section includes an overview of the interconnectedness of human and animal pathogens for several timely One Health events and describes the emergence of influenza viruses across the species barrier; rabies control; foodborne diseases and transmission among humans, animals, and plants; environmental reservoirs of cholera; and the role of human activity on the spread of white-nose syndrome in bats. The rest of the book is devoted to causes behind the emergence of antimicrobial drug resistance and the need for disease surveillance that can identify pathogens crossing animal–human interfaces to provide early warning of new public health challenges and describes new technologies and approaches for public health surveillance, and the challenge of making One Health a reality. The editors share several examples of successful applications of the One Health approach to highlight the impact of collaboration between human, animal, and environmental health partners. Remaining challenges of implementing a One Health approach are also presented in the context of thwarting the threat of emerging infectious diseases. Throughout the book, the editors provide case histories of notable recent zoonotic infections, which provide real-world examples of implementing a One Health approach for diseases such as West Nile virus disease, hantavirus, Lyme disease, and severe acute respiratory syndrome.

The availability of texts describing the One Health approach is important, and this book provides a concise overview of One Health from the infectious disease perspective. Although this book focuses on the role of One Health specifically for emerging infectious and zoonotic diseases, it is a valuable introduction to the field of One Health. The book applies to a wide audience—physicians, veterinarians, environmental scientists, public health officials, policy makers, and students. It is a useful resource for those who want a better understanding of One Health and their role in the One Health movement. The One Health concept continues to gain recognition as a critical tool to address public health issues at the animal–human-ecosystem interface to have the biggest impact on improving health for all. In the words of the editors, “the One Health approach is simply too important to ignore.”

